# Retrovesical Hydatid Cyst

**Published:** 1917-07

**Authors:** 


**Retrovesical Hydatid Cyst.** Potherat, Le Progres Med., May 19, page 170, reports a case in a soldier, aged 26, cured by operation. Attention was called to the cyst by sudden urinary retention, the diagnosis being enlarged prostate.

